# Spatiotemporal dynamics of cholera hotspots in the Democratic Republic of the Congo from 1973 to 2022

**DOI:** 10.1186/s12879-024-09164-9

**Published:** 2024-03-28

**Authors:** Nadège Taty, Didier Bompangue, Sandra Moore, J. J. Muyembe, Nancy Meschinet de Richemond

**Affiliations:** 1 Department of Infectious Disease Ecology and Control, Faculty of Medicine, University of Kinshasa, Kinshasa, Democratic Republic of the Congo; 2 Montpellier Geography and Spatial Planning Laboratory, Paul Valéry Montpellier 3 University, Montpellier, France; 3 National Program for the Elimination of Cholera and the Fight against Other Diarrheal Diseases, Ministry of Health, Hygiene and Prevention, Kinshasa, Democratic Republic of the Congo; 4 Chrono-Environment Laboratory, UMR 6249, University of Bourgogne Franche-Comté, Besançon, France; 5 Veolia Foundation, Aubervilliers, France; 6 National Institute of Biomedical Research, Kinshasa, Democratic Republic of the Congo

**Keywords:** Democratic Republic of the Congo, Cholera, Spatiotemporal outbreak dynamics, Cholera hotspots, Cholera outbreaks, Cholera epidemics, *Vibrio cholerae*

## Abstract

**Background:**

Since the early 1970s, cholera outbreaks have been a major public health burden in the Democratic Republic of Congo (DRC). Cholera cases have been reported in a quasi-continuous manner in certain lakeside areas in the Great Lakes Region. As these cholera-endemic health zones constitute a starting point for outbreaks and diffusion towards other at-risk areas, they play a major role in cholera dynamics in the country. Monitoring the spatiotemporal dynamics of cholera hotspots and adjusting interventions accordingly thus reduces the disease burden in an efficient and cost-effective manner.

**Methods:**

A literature review was conducted to describe the spatiotemporal dynamics of cholera in the DRC at the province level from 1973 to 1999. We then identified and classified cholera hotspots at the provincial and health zone levels from 2003 to 2022 and described the spatiotemporal evolution of hotspots. We also applied and compared three different classification methods to ensure that cholera hotspots are identified and classified according to the DRC context.

**Results:**

According to all three methods, high-priority hotspots were concentrated in the eastern Great Lakes Region. Overall, hotspots largely remained unchanged over the course of the study period, although slight improvements were observed in some eastern hotspots, while other non-endemic areas in the west experienced an increase in cholera outbreaks. The Global Task Force on Cholera Control (GTFCC) and the Department of Ecology and Infectious Disease Control (DEIDC) methods largely yielded similar results for the high-risk hotspots. However, the medium-priority hotspots identified by the GTFCC method were further sub-classified by the DEIDC method, thereby providing a more detailed ranking for priority targeting.

**Conclusions:**

Overall, the findings of this comprehensive study shed light on the dynamics of cholera hotspots in the DRC from 1973 to 2022. These results may serve as an evidence-based foundation for public health officials and policymakers to improve the implementation of the Multisectoral Cholera Elimination Plan, guiding targeted interventions and resource allocation to mitigate the impact of cholera in vulnerable communities.

**Supplementary Information:**

The online version contains supplementary material available at 10.1186/s12879-024-09164-9.

## Background

Cholera is an infectious diarrheal disease caused by pathogenic strains of *Vibrio cholerae*, a gram negative bacterium [1]. Humans can become infected through ingestion of contaminated water or food or direct human-to-human contact [2]. After a short incubation period of a few hours to 10 days, disease symptoms can vary from asymptomatic; mild diarrhea, which affects approximately 10% of people exposed; to severe diarrhea affecting 1% of cases [2].

Cholera remains a major public health issue globally. In 2022, a total of 472,697 cases and 2349 deaths (case-fatality rate of 0.5%) have been reported worldwide. In 2022, the total number of cases notified to the World Health Organization (WHO) was twice that reported in 2021, with 44 countries reporting cases in 2022 compared with 35 countries reporting cases in 2021.

In 2022, with 18,961 suspected cases, the Democratic Republic of the Congo (DRC) ranked fourth in the world and second in Africa after Nigeria [3]. Indeed, the DRC has been heavily burdened by cholera for the past several decades [4, 5]. During the current cholera pandemic, the disease was first introduced into the DRC in 1973 from the west in Kongo Central Province and then in 1978 from the east in Tanganyika Province [3, 5, 6]. Since 1994, cholera has been endemic in the Great Lakes Region of eastern DRC [7]. The eastern provinces of Ituri, North Kivu, South Kivu, Tanganyika, Haut Lomami and Haut Katanga have been identified as cholera endemic [8]. Meanwhile, epidemics have only sporadically occurred in the western part of the country [7].

Studies conducted in the DRC in 2007 have shown that cholera cases are reported in a quasi-continuous manner in certain lakeside health zones in the eastern Great Lakes Region [7]. Cholera is considered endemic in these health zones as they constitute a starting point for cholera outbreaks and diffusion towards other at-risk areas and consequently play a major role in cholera dynamics in the country. Monitoring the spatiotemporal dynamics of cholera hotspots and adjusting interventions accordingly thus reduces the cholera burden in an efficient and cost-effective manner. Cholera-endemic health zones were classified using a method that considers both epidemiological (e.g., incidence and persistence) and environmental parameters (e.g., proximity to lakes). In 2008, prevention and control interventions were prioritized in these endemic health zones in the DRC’s first strategic *Multisectoral Cholera Elimination Plan* (MCEP) [9]. To date, three versions of the country’s MCEP have been implemented, and the list of targeted cholera-endemic health zones has been updated every 5 years [10, 11]. The fourth plan was validated in November 2022 and covers the period 2023–2027.

Previous approaches to efficiently control infectious diseases have been based on the concept of identifying heavily-affected areas (or “hotspots”) for targeted interventions [12], as shown in the strategic plans to control epidemics of Ebola virus disease [13], malaria [14] and cholera [11]. Cholera hotspot mapping was also integral to control the disease in the Mediterranean region in 2015 [12].

Ten years after the 2007 study in the DRC, the Global Task Force on Cholera Control (GTFCC) published the global roadmap “Ending Cholera”, which aims to reduce cholera deaths by 90% and eliminate cholera transmission by 2030 [15]. The GTFCC advocates a broader definition of cholera hotspots as specific and relatively small areas where the cholera burden is most concentrated and that play a central role in the spread of the disease. To facilitate the development of national cholera elimination plans, the GTFCC has recently established a tool to identify and classify cholera hotspots. Accordingly, hotspots are classified into three priority levels (high, medium and low priority) based on two epidemiological indicators: average annual incidence of cholera cases per 100,000 inhabitants and cholera persistence over a five-year period [16]. However, this method does not consider the local environmental factors that drive cholera dynamics in each area. The GTFCC had disseminated this algorithm to countries to guide the identification of cholera hotspots to be used in National Cholera Elimination Plans.

The concept of targeting interventions in specific areas that play a role in outbreak dynamics to prevent cholera is widely accepted; however, the definition of hotspots and the method used to identify and classify these areas have yet to be standardized [7, 17].

The current study aimed to describe the spatiotemporal cholera dynamics in the DRC from 1973 to 2022. We describe the cholera burden in the DRC at the province level from 1973 to 1999. To optimize cholera elimination interventions, we also identified and classified cholera hotspots at the provincial and health zone levels from 2003 to 2022 and described the spatiotemporal evolution of hotspots. We applied and compared three different classification methods to ensure that cholera hotspots are identified and classified according to the DRC context.

## Methods

### Study site

We conducted a descriptive cross-sectional study of cholera outbreaks in the DRC from 1973 to 2022. The country is divided administratively into 26 provinces and 519 health zones. In 2020, the estimated population of the DRC was 93,852,092 inhabitants distributed in a heterogeneous manner [18], with an average of > 50 inhabitants/km^2^ in the eastern Great Lakes Region and 5–10 inhabitants/km^2^ in western DRC [19]. The population density is lowest in the center of the country [20]. Since 1994, the Congolese part of the Great Lakes Region has been one of the most unstable regions in Africa in terms of insecurity and complex crisis [21].

The DRC is located on both sides of the equator, with the Atlantic coast in the west and the Great Lakes Region in the east [20]. The largest lakes in the DRC are located in the east (e.g., Lakes Tanganyika, Kivu, Moero, Edward and Albert). In the center of Haut Lomami Province, there are many small lakes around the Congo River basin, the largest of which is Lake Upemba in Bukama Health zone [21] (Additional file 1).

### Data collection

#### Information on cholera outbreaks recorded in the DRC before the surveillance system was implemented (1973–1999)

We conducted a literature review of cholera outbreaks documented from 1973 to 1999 (i.e., before the Integrated Disease Surveillance and Response System (IDSR) was established in the DRC in 2000) using PubMed, Google scholar and Google queries. The following keywords were used: (“cholera” AND “Democratic Republic of Congo”); (“cholera” AND “ Zaire “); and (“cholera” AND “Africa” AND “1973″). Peer-reviewed and non-peer-reviewed articles from the period 1973–1999 were assessed. Unpublished mission reports describing cholera outbreaks in the various provinces of the DRC were also included in the analysis.

#### Cholera data recorded from 2000 to 2022

In the DRC, the IDSR reports weekly suspected cases of diseases with epidemic potential, including cholera, at the province and health zone levels. We assessed epidemiological data on suspected cholera cases reported in the DRC from 2000 to 2022. The IDSR uses two definitions of suspected cholera cases depending on whether a cholera outbreak has been declared by the Ministry of Health. In areas without a declared cholera outbreak, a suspected cholera case is any patient 2 years of age or older with acute watery diarrhea and severe dehydration or dying of acute watery diarrhea. In areas where a cholera outbreak has been declared, a suspected cholera case is any person with or dying from acute watery diarrhea [22].

#### Population data

Population estimates for the health zones and provinces from 2000 to 2022 were used based on data obtained from the Expanded Program on Immunization applying a stable population growth rate of 1.03.

#### Definitions

- Cholera-endemic health zones: A cholera-endemic lakeside area that notifies cholera cases in a quasi-continuous manner, with lull periods (reporting zero cases per week) of < 16 weeks (source: the DRC Department of Ecology and Infectious Disease Control (DEIDC); MCEP 2023–2027) [23].
- Cholera-endemic province: a province containing cholera-endemic health zones.
- Cholera hotspot: A geographically-limited area where cholera persists or reoccurs regularly, thus playing a central role in the spread of the disease to other areas [24].
- Cholera non-endemic area: An area that reports cholera cases in a recurrent (> 3 outbreaks in the previous 5 years), intermittent (< 3 outbreaks in the previous 5 years) or sporadic (1 outbreak in the previous 5 years) manner.
- Metastable cholera area: A lakeside area (that is not considered a cholera-endemic health zone) that exhibits an intermediate phase between the endemic and non-endemic phases with lull periods (reporting zero cases per week) of > 16 weeks. These zones can evolve into a non-endemic phase (if conditions improve) or an endemic phase (if corrective actions are not effective).
- Annual lull period: The period of seven consecutive weeks with the lowest number of cholera cases reported in a given year.
- Cholera residual zone: An area where cholera cases persist after an outbreak (during annul lull periods).
- Cholera outbreak:
- In non-endemic provinces or health zones: at least 1 laboratory-confirmed cholera case or an increase in the number of suspected cases for > 3 consecutive weeks [25].
- In endemic provinces and health zones: an increase in the number of suspected cholera cases (above the local endemicity threshold) for at least 3 consecutive weeks [25].

### Data analysis

#### Data pre-processing and organization

Data for the period 1973 to 1999 were organized in an Excel file with the following variables: province, health zone, year, and number of cholera outbreaks reported. The annual sum of outbreaks from 1973 to 1999 was then calculated.

For the analysis of data from 2000 to 2022, the data series were subdivided into four periods of 5 years (corresponding to the implementation period of each national cholera plan): 2003–2007 (the period before the plan was established), 2008–2012 (1st *MCEP*), 2013–2017 (2nd *MCEP*) and 2018–2022 (3rd *MCEP*).

#### Identification and classification of hotspots

The spatiotemporal dynamics of cholera hotspots were analyzed at the province and health zone levels by dividing the study period into four five-year sub-periods, i.e., [1] 2003–2007, [2] 2008–2012, [3] 2013–2017 and [4] 2018–2022. These sub-periods correspond to the period before the first MCEP was implemented (2003–2007) and the first three MCEPs in the DRC (2008–2012, 2013–2017 and 2018–2022). Three methods were applied to identify and classify cholera hotspots in the DRC:

1. Identification of spatiotemporal clusters of cholera cases: We used Kulldorff’s retrospective spatiotemporal permutation scan statistic to detect spatiotemporal clusters of cholera cases using SaTScan version 9.6 [26]. Clusters were detected at the province level if the area concerned had a higher proportion of cases than the other areas during a given week. The most likely statistically significant cluster (window of highest probability) was estimated for each random permutation by 999 Monte Carlo replications of the simulated data set under the null hypothesis. We set the maximum spatial window to a radius of 125 km after identifying large spatiotemporal clusters containing many statistically significant subclusters in preliminary analyses [27, 28].
2. GTFCC cholera hotspot classification method: Using this method, cholera hotspots were identified based on two indicators: [1] the average attack rate per 100,000 inhabitants and [2] the proportion of weeks during which cases were notified over the course of a five-year period. The threshold for the average attack rate and the proportion of weeks with cases reported were set at 50%. Hotspots were then classified according to three priority levels: high, moderate and low priority [29].
3. The DEIDC cholera hotspot classification method: The weekly time series of cholera cases for each year were analyzed to identify annual lull periods (e.g., a period of seven consecutive weeks with the lowest number of cholera cases reported in a given year). Annual lull periods were defined by first identifying the week with the fewest cholera cases in a given year (reference week). Next, the 3 weeks before and after the reference week were included to define the annual lull period of 7 weeks. We then identified provinces and health zones that reported cholera cases during these lull periods. For each five-year sub-period, we calculated the number of years that a health zone or province reported residual cholera cases during the annual lull periods. Only provinces or health zones that reported residual cholera cases for at least two of the 5 years were considered “residual areas”. To categorize the provinces and zones, the evolution of weekly cholera cases was analyzed at the province and health zone levels over a five-year period. Lull periods of at least 8 weeks were considered to categorize the provinces and health zones into hotspots of type A, B or C (Table 1).

**Table 1:** Characteristics of cholera hotspots according to the DEIDC method

**Hotspot type**	**Lake area**	**Cholera-endemic health zone**	**Cholera outbreak profile**	**Duration of lull periods (notification of 0 cases)**
A	Yes	Yes	Endemic	< 8 weeks
B1				8–16 weeks
B2		No	Metastable	16–24 weeks
B3				24 weeks – 5 years
				**Number of outbreaks over last 5 years**
C1	No	No	Non-endemic (recurrent outbreaks)	≥ 3 outbreaks
C2			Non-endemic (intermittent outbreaks)	< 3 outbreaks
Non-HS			Non-endemic (sporadic outbreaks)	1 outbreak
Non-HS				0 outbreaks

*Non-HS* Non-hotspot area

#### Hotspot evolution score

The evolution in Type A and B hotspots (at the health zone level) was calculated using a coefficient of 8 as follows: non-hotspots = 8, B3 = 16, B2 = 24, B1 = 32 and A = 40. The rate of change was calculated as followings: rate of change = (final value - initial value)/initial value*100 [30].

#### Cartography

Maps were produced using QGIS 3.16 Madeira software with shapefiles obtained from www.DIVA-GIS gis.org/gdata corresponding to provinces and health zones in the DRC.

#### Other analysis software

The databases were cleaned and analyzed for weekly cases and deaths (cholera time series) using Microsoft Excel and R software.

## Results

### Overview of provinces in the DRC affected by cholera outbreaks from 1973 to 1999

Cholera was introduced into the DRC twice during the 1970s. In western DRC, cases were first reported in 1973 in Kongo Central Province and then rapidly spread to Kinshasa. Other western provinces along the Congo River (e.g., Mai Ndombe, Equateur) were later affected. In eastern DRC, cases were first imported in September 1977 in Kalemie, in Tanganyika Province, from Kigoma in Tanzania. The outbreak then rapidly spread to all eastern provinces in the Rift Valley (e.g., Haut-Katanga, Haut-Lomami, North Kivu, South Kivu and Ituri). From 1973 to 1999, the two entry-point provinces (Kongo Central and Tanganyika) were affected the most often (five times each), followed by North Kivu and Haut Katanga (four times each) (Additional file 2).

### Classification of cholera hotspots in the DRC from 2003 to 2022

**Fig. 1:**
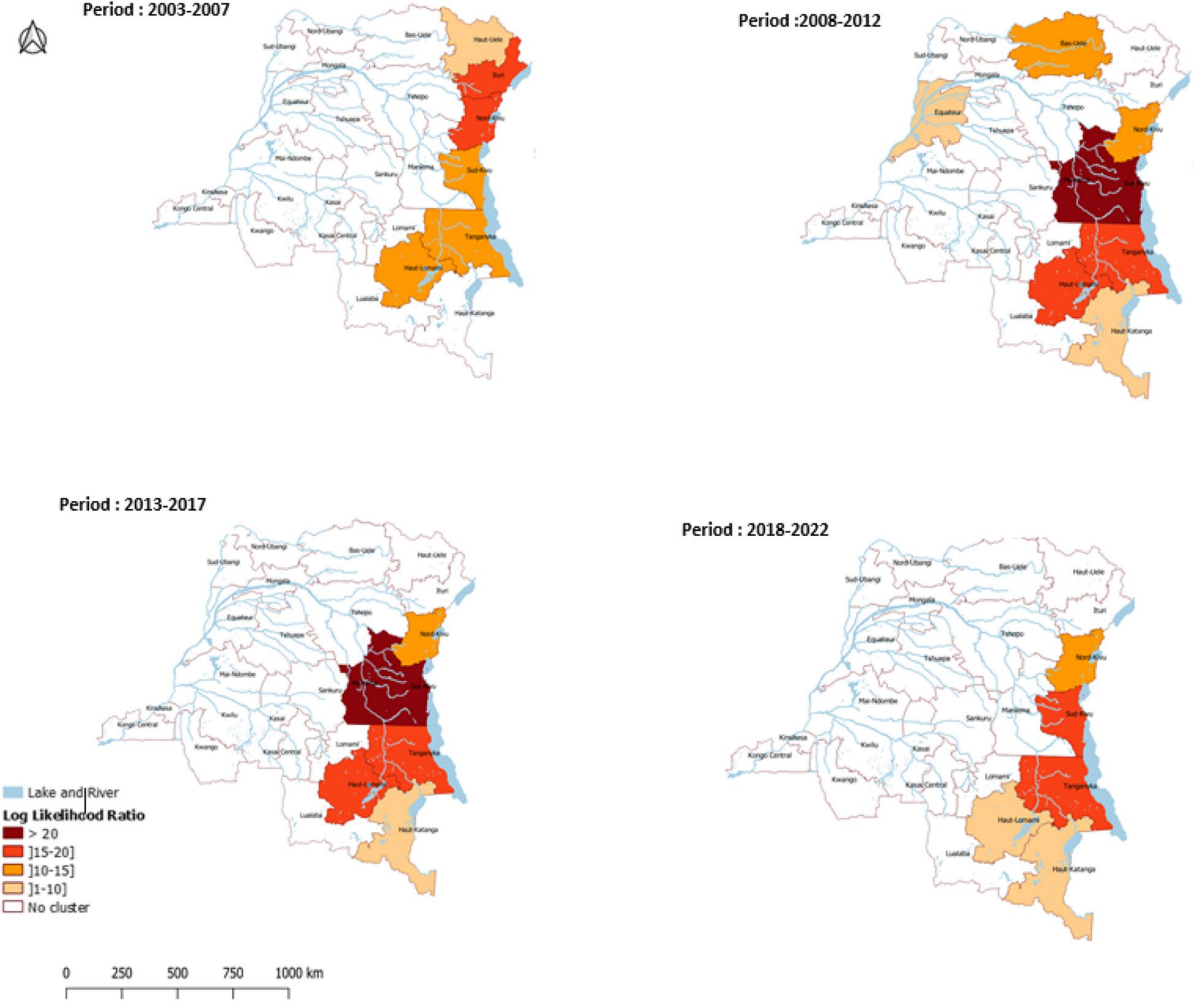
Classification of cholera hotspots in the DRC at province level (2003–2022): cluster method

The cluster method was applied to identify hotspots at the province level. All high-risk clusters were located in the east during each period except 2008–2012. In 2008–2012, the provinces of Bas-Uele and Équateur were also identified as cholera clusters. During the period 2003–2007, the provinces most at risk were North Kivu and Ituri. In the following two periods (2008–2017), the provinces most at risk were Maniema and South Kivu, followed by Tanganyika and Haut-Lomami. In the recent period 2018–2022, the provinces most at risk were South Kivu and Tanganyika. Overall, the highest risk gradually shifted southward over the course of the study period. Furthermore, high-risk provinces were often flanked to the north and south by medium-risk provinces (Fig. 1).

**Fig. 2:**
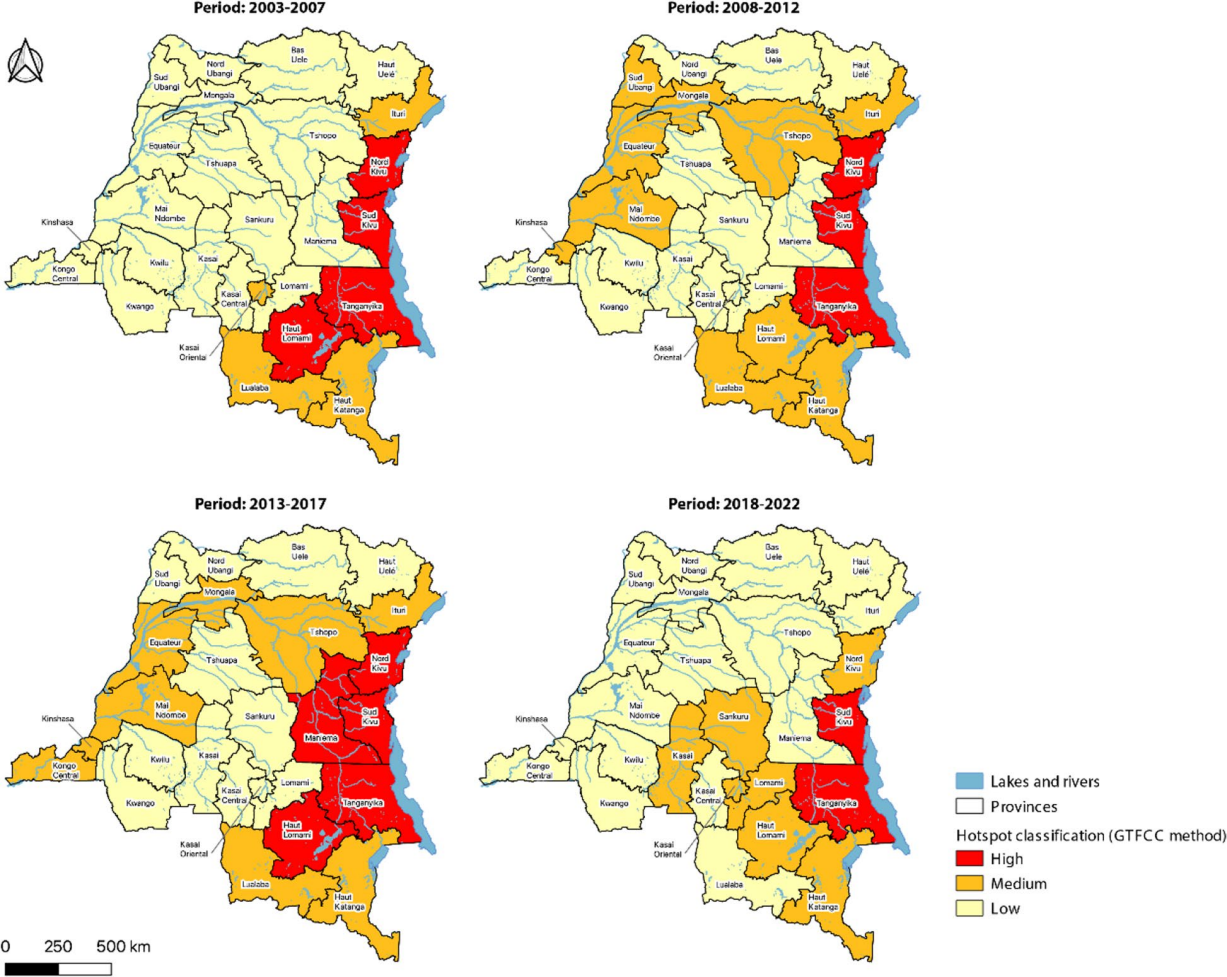
Classification of cholera hotspots in the DRC at province level (2003–2022): GTFCC method

Using the GTFCC method to identify hotspots at the province level, cholera-endemic provinces were classified as medium- or high-priority hotspots during each period, except for the 2018–2022 period, during which Ituri Province was not identified as a hotspot. Only cholera-endemic provinces were identified as high-priority hotspots, with the exception of Maniema Province, which was also identified as a high-priority hotspot during the 2013–2017 period. Several provinces along the Congo River were also identified as medium-priority cholera hotspots during the 2008–2012 and 2013–2017 periods, while from 2018 to 2022, central provinces (e.g., Kasai, Sankuru, Kasai Oriental) were also identified as medium-priority cholera hotspots (Fig. 2).

**Fig. 3:**
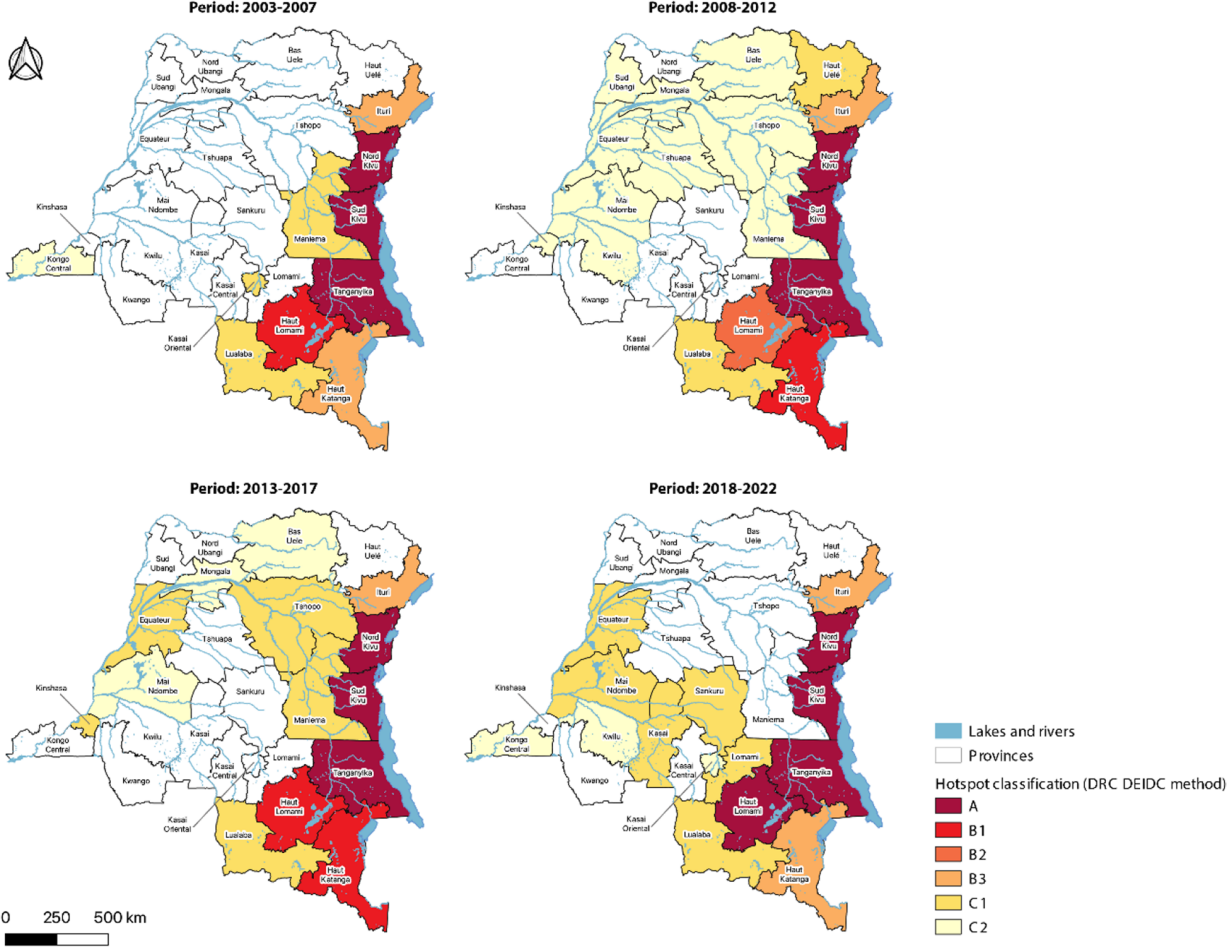
Classification of cholera hotspots in the DRC at province level (2003–2022): DEIDC method

Using the DEIDC method to identify hotspots at the province level, the three provinces of North Kivu, South Kivu and Tanganyika were classified as Type A hotspots during each period. The province of Haut-Lomami was classified as Type B1 during two periods (2003–2007 and 2013–2017) and then transitioned to a type A hotspot during the last period (2018–2022). Haut Katanga was also a Type B1 hotspot during the periods 2008–2012 and 2013–2017. Low-risk cholera hotspots (C1-C2) were identified along the Congo River during the 2008–2012 and 2013–2017 periods, which corresponds to the periodic westward spread of cholera epidemics in 2011–2012 and 2015, respectively. During the 2018–2022 period, hotspots remained in western DRC and were also identified further south (Fig. 3).

Comparing the classification results between the GTFCC and DEIDC methods, the Type A or B hotspots according to the DEIDC method were nearly always classified as “high-priority” provinces according to the GTFCC method. Similarly, Type C1 or C2 hotspots (DEIDC method) were often classified as “low-priority” provinces according to the GTFCC. However, the provinces classified as “medium-priority” according to the GTFCC, displayed a wide variety of profiles according to the DEIDC method ranging from Type A to Type C2 (Table 2).

**Table 2:**
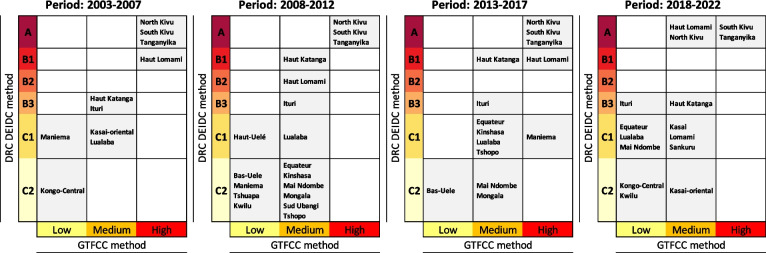
Comparison of cholera hotspots in the DRC at the province level using the GTFCC and DEIDC methods

**Fig. 4:**
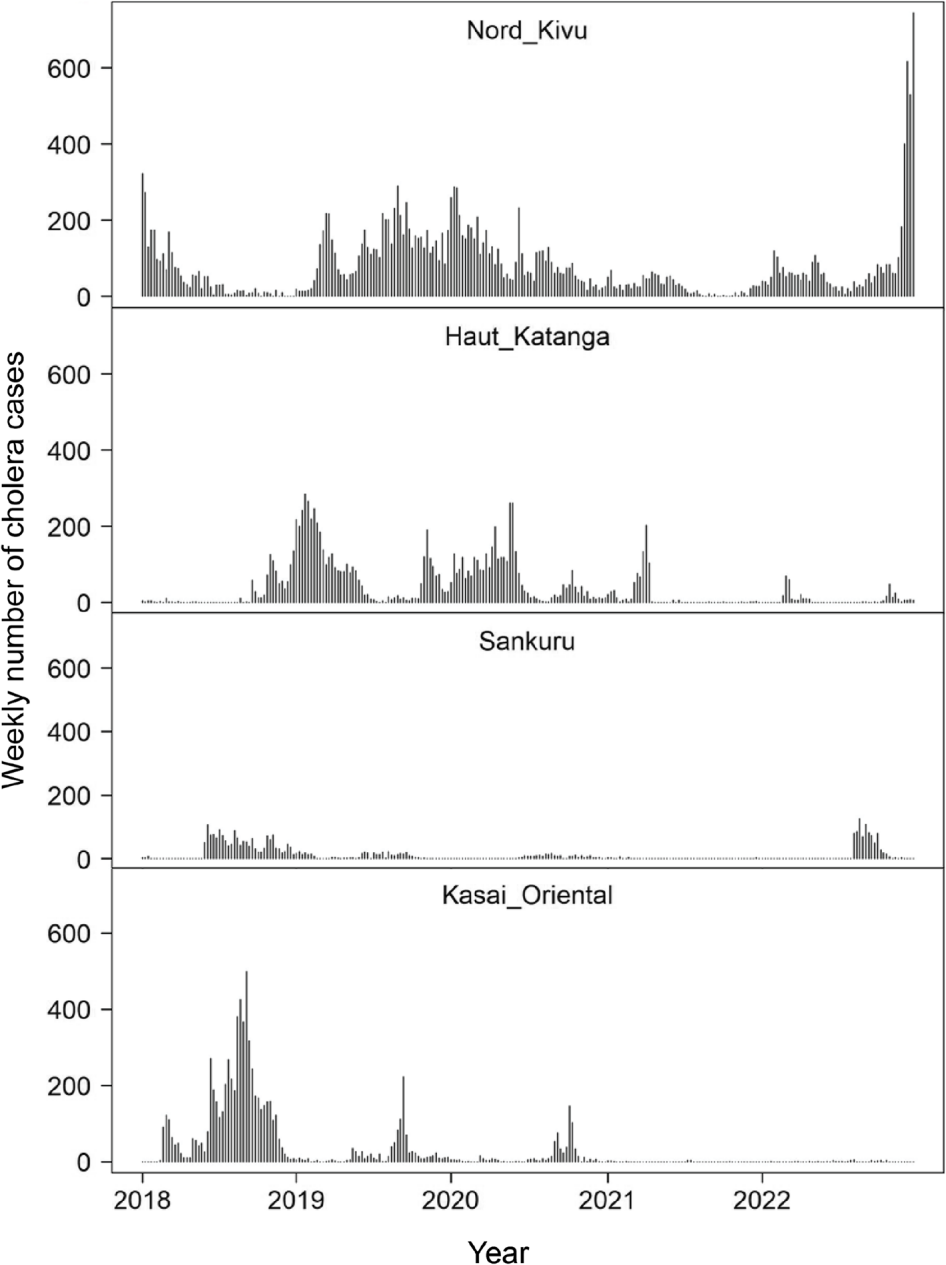
Weekly number of cholera cases in medium-priority provinces according to GTFCC method (2018–2022)

To further assess the variation regarding the medium-priority hotspots using the GTFCC method, a detailed assessment was conducted on the weekly cholera-case time series for four medium-priority provinces during the period 2018 to 2022 (e.g., Sankuru, Kasaï-Oriental, North Kivu and Haut-Katanga). According to the DEIDC method, these provinces had four distinct hotspot profiles: North Kivu (Type A), Haut-Katanga (Type B3), Sankuru (Type C1) and Kasaï-Oriental (Type C2). Indeed, comparing each time series indicates a clear difference in epidemic profile (i.e., continuous weekly cases in North Kivu, short lull periods in Haut-Katanga and extended lull periods in Sankuru and Kasaï-Oriental) (Fig. 4).

**Fig. 5:**
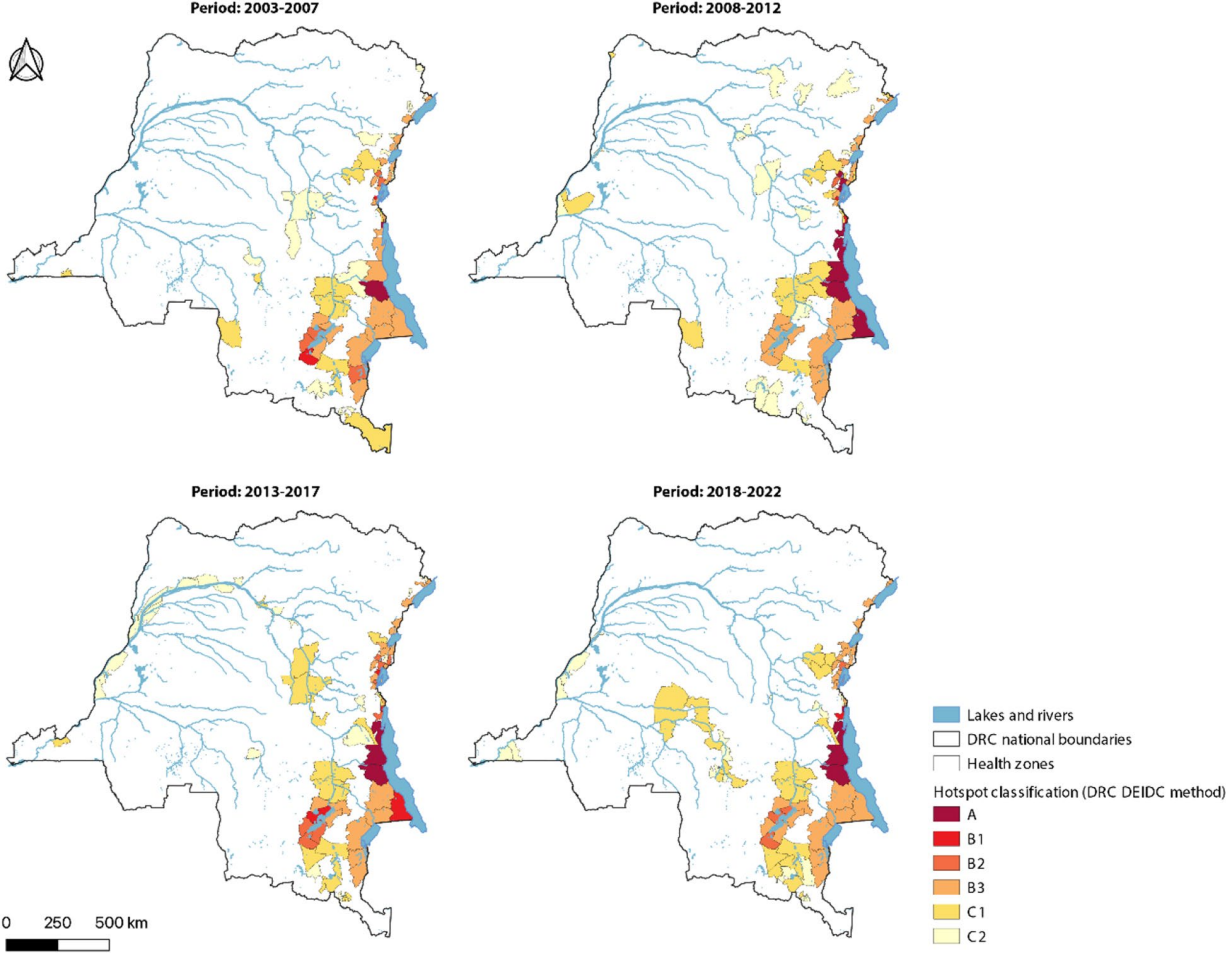
Classification of cholera hotspots in the DRC at health zone level (2003–2022): DEIDC method

As the DEIDC method integrates both the environmental and epidemiological profile of each area, hotspots were then assessed at the health zone level using the DEIDC method. The high-risk hotspots (Type A and B) were located in the lake region in the east of the country. From 2003 to 2007, cholera hotspots were primarily located in the eastern lakeside region of the country. From 2008 to 2012, cholera hotspots spread to the west of the country, starting with health zones along the Congo River. This pattern continued in 2013–2017. From 2018 to 2022, health zones in the center of the country along the Kasaï River and its tributaries were also identified as cholera hotspots (Fig. 5).

Comparing the most recent two periods (2013–2017 and 2018–2022), we observed a decrease in Type A-B1 hotspots and a slight increase in Type B2-C2 hotspots. However, the overall number of each hotspot type largely returned to the baseline over the entire study period, with an increase in Type C1 hotspots (Table 3).

**Table 3:** Number of cholera hotspots at the health zone level from 2003 to 2022 (DEIDC method)

Type of hotspot	Number of hotspots per period			
	2003–2007	2008–2012	2013–2017	2018–2022
A (Endemic)	4	11	4	4
B1 (Endemic)	2	2	6	2
**Total A et B1**	**6**	**13**	**10**	**6**
B2 (Metastable)	5	2	10	6
B3 (Metastable)	31	29	26	32
**Total B2 et B3**	**36**	**31**	**36**	**38**
C1 (Non-endemic)	18	17	23	31
C2 (Non-endemic)	26	19	26	21
**Total C**	**44**	**36**	**49**	**52**

From 2003 to 2022, a total of 46 health zones, or 8.8% of the 519 zones in the DRC, were identified as Type A and B hotspots, including 12 in North Kivu, 13 in South Kivu, 4 in Tanganyika, 4 in Haut-Katanga, 8 in Haut-Lomami and 5 in Ituri. From 2003 to 2022, these health zones reported 62.8% of all cases (298,780 of a total of 475,327 cholera cases). Three health zones were not identified as cholera hotspots during the period 2003 to 2007 but eventually transitioned to hotspots: Kahele (South Kivu) since 2008 as well as Kibirizi (North Kivu) and Mulongo (Haut-Lomami) since 2013. Only two health zones identified as cholera hotspots during 2003–2007 transitioned to non-hotspot status during the 2018–2022 period: Rethy in Ituri and Kyondo in North Kivu (Table 4).

**Table 4:**
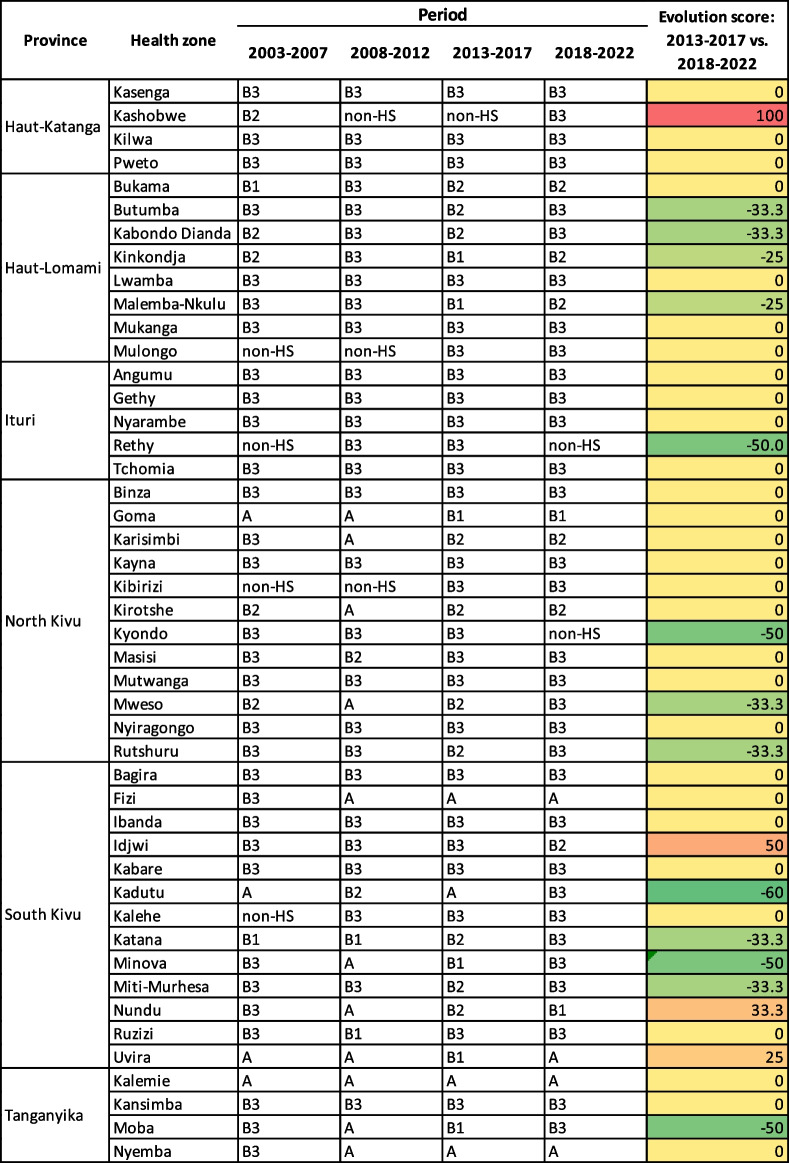
Evolution of Type A and B hotspots at the health zone level

*Non-HS* Non-hotspot area

Overall, comparing the periods 2013–2017 and 2018–2022, the majority of hotspots maintained the same profile (29 health zones, 63% of type A/B hotspots). During this time, the situation improved in 13 hotspots (28.3% of type A/B hotspots). Meanwhile, four health zones transitioned to a higher-risk hotspot status (8.7% of type A/B hotspots) (e.g., Idjwi, Nundu, Uvira and Kashobwe) (Table 4).

## Discussion

This comprehensive study employed several analysis approaches to identify and classify cholera hotspots in the DRC from 2003 to 2022. The results reveal trends in cholera hotspot dynamics at both the province and health zone levels. High-priority hotspots were concentrated in the eastern Great Lakes Region, according to all three methods. Overall, hotspots largely remained unchanged over the course of the study period, although slight improvements were observed in some hotspots in endemic provinces, while other non-endemic provinces experienced an increase in cholera outbreaks. The GTFCC and DEIDC methods largely yielded similar results for the high-risk hotspots. However, the medium-priority hotspots identified by the GTFCC method were further sub-classified by the DEIDC method, thereby providing a more detailed ranking for priority targeting.

To identify the most adapted approach to classify hotspots in the DRC, we compared the results of the GTFCC and DEIDC methods [31–33]. The GTFCC tool is used to classify cholera hotspots into three priority classes (high, moderate and low) based on two indicators: the average annual incidence of cholera cases per 100,000 inhabitants and cholera persistence [29]. Meanwhile, the DEIDC method considers multiple epidemiological indicators as well as environmental factors that play a key role in cholera dynamics (as detailed in Table 1). Comparing the classification results, the Type A or B hospots according to the DEIDC method were identified as “high-priority” provinces by the GTFCC method. However, the DEIDC method was more sensitive and specific. Provinces identified as “medium-priority” using the GTFCC method were further sub-classified using the DEIDC method (ranging from Type A to C2 hotspots), thus highlighting the need for a more detailed sub-classification of GTFCC medium-priority hotspots. Indeed, assessment of the weekly cholera-case time series for four “medium-priority” provinces during the 2018–2022 period indicates distinct epidemiological profiles (ranging from continuous reporting of weekly cases to periodic epidemics with extended lull periods). The more detailed classification of these high- and medium-priority hotspots provided by the DEIDC method enables limited resources to target the most at-risk areas, which are the source of outbreak onset and diffusion. This sub-classification also enables public health officials to assess the progress in hotspots when monitoring cholera elimination programs.

These results highlight the trends in cholera hotspot dynamics over the past two decades. High-risk areas were located in the eastern Great Lakes Region of the country over the entire study period, as observed in previous studies in the DRC [7, 25]. Lower-risk hotspots were associated with spread outside of high-risk endemic areas. Indeed, both province- and health zone-level analyses demonstrate the westward spread of cholera risk along the Congo River during the periods 2008–2012 and 2013–2017. Over the course of the study period, province-level Type A/B hotspots remained largely unchanged, with the exception of Haut Lomami, which gradually transitioned from Type B1/B2 to Type A. However, Type C hotspots increased over time, which is in line with a previous study noting an increase in cases in non-endemic provinces [34]. The improvement in endemic areas may be due to the implementation of several emergency and development projects in hotspots in eastern DRC by various players such as VEOLIA, the French Development Agency, UNICEF and WHO [34].

From 2003 to 2022, a total of 46 health zones (8.8% of the 519 health zones in the DRC) were identified as Type A and B hotspots. These lake-side health zones reported 62.8% of all cases (total of 475,327 cholera cases recorded). Comparing the most recent two periods (2013–2017 and 2018–2022), the majority of Type A-B hotspots maintained the same profile. Factors associated with continued outbreaks in eastern endemic provinces include socio-political unrest and environmental conditions [35]. Conflict can further drive cholera diffusion and hinder outbreak response efforts by disrupting basic services (including water, sanitation and hygiene (WASH)) and healthcare systems [36, 37]. Conflict can also worsen or cause population displacement, thus further rendering populations vulnerable to disease outbreaks [37]. A recent study has found that conflict significantly increased the rate of cholera outbreaks the DRC, especially in Tanganyika, Kasaï-Oriental, Maniema, North Kivu and Kasaï [38]. Furthermore, 12.3% of outbreaks in the DRC from January 1997 to May 2020 were attributable to conflict [38]. A separate study has also found that increased cholera case numbers, conflicts, and internally displaced persons in endemic areas in the east were associated with an increased risk of cholera diffusing outside of eastern provinces [39]. Regarding environmental conditions in eastern provinces, most of the lakes in eastern DRC are of tectonic origin, with physico-chemical properties conducive to the survival of *Vibrio cholerae* [40]. Thus, an aquatic environmental reservoir in the east with physico-chemical conditions that support toxigenic forms of *Vibrio cholerae* [41], combined with poor access to safe drinking water and proper sanitation facilities, may drive continuous exchange between humans and the environment [42]. This hypothesis of an environmental reservoir of *Vibrio cholerae* is also supported by previous ecological studies conducted in the DRC [7, 43]. Future studies on the genetic characterization of toxigenic *Vibrio cholerae* strains circulating in eastern DRC (both clinical and environmental isolates) could help to understand this phenomenon.

During the 2018–2022 period, the provinces of Kasai, Kasai Oriental, Sankuru and Lomami (located in the Grand-Kasaï Region) were identified as hotspots. These provinces had only previously reported epidemics in 2002–2003 [43] and then 13 years later in 2017. The outbreaks in 2017 may have been due to the persistence of outbreaks in certain areas of the west of the country following the east-west spread of cholera in 2015 [28]. The onset of cholera outbreaks in Grand-Kasaï Region during week 40, 2017 was linked to the poorly extinguished outbreaks in Bandundu (Kwilu) and the Bolobo area (Mai-Ndombe) that spread following the return of displaced people from the ethnic unrest of the “Kamuena-Sampu” rebellion [44]. The epidemic has since persisted in this region until at least 2023. Significant challenges to control the epidemic in this region have been due to poor access to water and sanitation in addition to limitations in outbreak response (reduced response at the end of outbreaks, lack of outbreak preparedness or prevention activities, and insufficient supporting partners and capacity).

Some study limitations should be noted. The data on suspected cholera cases may not reflect the real cholera burden in the DRC, as these data only take into account patients who consulted health facilities. Furthermore, although outbreaks are initially confirmed via *Vibrio cholerae* culture, subsequent cases are based on clinical criteria. Epidemiological surveillance in the eastern provinces of the DRC (e.g., North Kivu Province) is also relatively weak due to socio-political unrest and insecurity. The DEIDC method identifies hotspots based on lull periods and the number of outbreaks, which are determined based on the weekly notification of suspected cholera cases. Ideally, each outbreak should be confirmed using molecular analyses of the *Vibrio cholerae* strains in circulation [45]. Nevertheless, a study conducted in the DRC has shown that data on suspected cholera cases can be used for epidemiological or health research [46]. Finally, this study did not take into account other factors that can influence cholera dynamics, such as WASH, socio-economic and other environmental factors.

These results highlight several potential measures to further strengthen cholera elimination efforts. First, a specific list of interventions per hotspot type should be established. For example, preventive vaccination campaigns could be planned in Type A-B health zones, while reactive vaccination is conducted in Type C health zones. Second, as previous studies have shown that major factors of cholera persistence include socio-economic and cultural factors [47], in-depth analyses of cholera risk factors in hotspots (e.g., WASH, socio-economic and environmental factors) [34] should be conducted. These factors could also be incorporated into future hotspot analyses. Third, all aspects of MCEP implementation should be monitored and documented, including coordination, management and funding. Fourth, the hotspot analysis should be regularly updated every 4 years to monitor hotspot evolution and adjust elimination activities accordingly. Regular updates would also serve as a tool to monitor MCEP progress. Finally, in key hotspots, a similar analysis should be conducted at a lower administrative level to better target control activities.

## Conclusions

In conclusion, the findings of this comprehensive study, which employed three distinct classification methods, shed light on the dynamics of cholera hotspots in the DRC from 1973 to 2022. From 1973 to 1999 in the DRC, the two cholera entry point provinces in the DRC (Kongo Central in the west and Tanganyika in the east) were the most affected in terms of frequency of cholera outbreaks; both provinces were affected five times. However, cholera only became endemic in the east of the country. High-priority hotspots were largely concentrated in the eastern Great Lakes Region. Overall, hotspots largely persisted over the course of the study period. From 2013 to 2017 to 2018–2022, we observed a slight decrease in hotspots in endemic areas, while some areas non-endemic for cholera experienced an increase in cholera outbreaks. Comparing the classification results, the GTFCC and DEIDC methods were largely in agreement for the high-risk hotspots. However, the DEIDC method provided a more detailed classification of the medium-priority hotspots identified by the GTFCC approach. Indeed, this limitation may explain the development of a new hotspot algorithm that has been recently published by the GTFCC but is not yet available at the country level, at least in the DRC. Going forward, to improve the cholera hotspot classification method, further analyses should include biological case-confirmation and risk factor data (e.g., WASH, socio-economic and environmental indicators). The hotspot analysis should be conducted regularly to monitor hotspot evolution, assess the effectiveness of MCEP implementation, and adjust activities accordingly. A package of activities according to hotspot type should be defined for efficient and effective elimination. Overall, these results serve as an evidence-based foundation for public health officials and policymakers to improve MCEP implementation, guiding targeted interventions and resource allocation to mitigate the impact of cholera in vulnerable communities.

### Supplementary Information

**Supplementary Material 1.**

**Supplementary Material 2.**

## Data Availability

The data are available from the authors upon reasonable request and with the permission of the Head of the Department of Infectious Diseases Ecology Didier Bompangue.

## Abbreviations

DEIDC: Department of ecology and infectious disease control
DRC: Democratic republic of the congo
GTFCC: Global task force on cholera control
IDSR: Integrated disease surveillance and response system
MCEP: Multisectoral cholera elimination plan
Non-HS: Non-hotspot
UNICEF: United nations international children’s emergency fund
WASH: Water, sanitation and hygiene
WHO: World health organization
