# Supplementary material for: Spatiotemporal dynamics of cholera hotspots in the Democratic Republic of the Congo from 1973 to 2022
Source: BMC Infect Dis. 2024 Mar 28;24:360. doi: 10.1186/s12879-024-09164-9 (PMC10976723; doi:10.1186/s12879-024-09164-9)
Supplement: Supplementary file 1 — Supplementary Material 1. [file 12879_2024_9164_MOESM1_ESM.docx]

Additional file 1


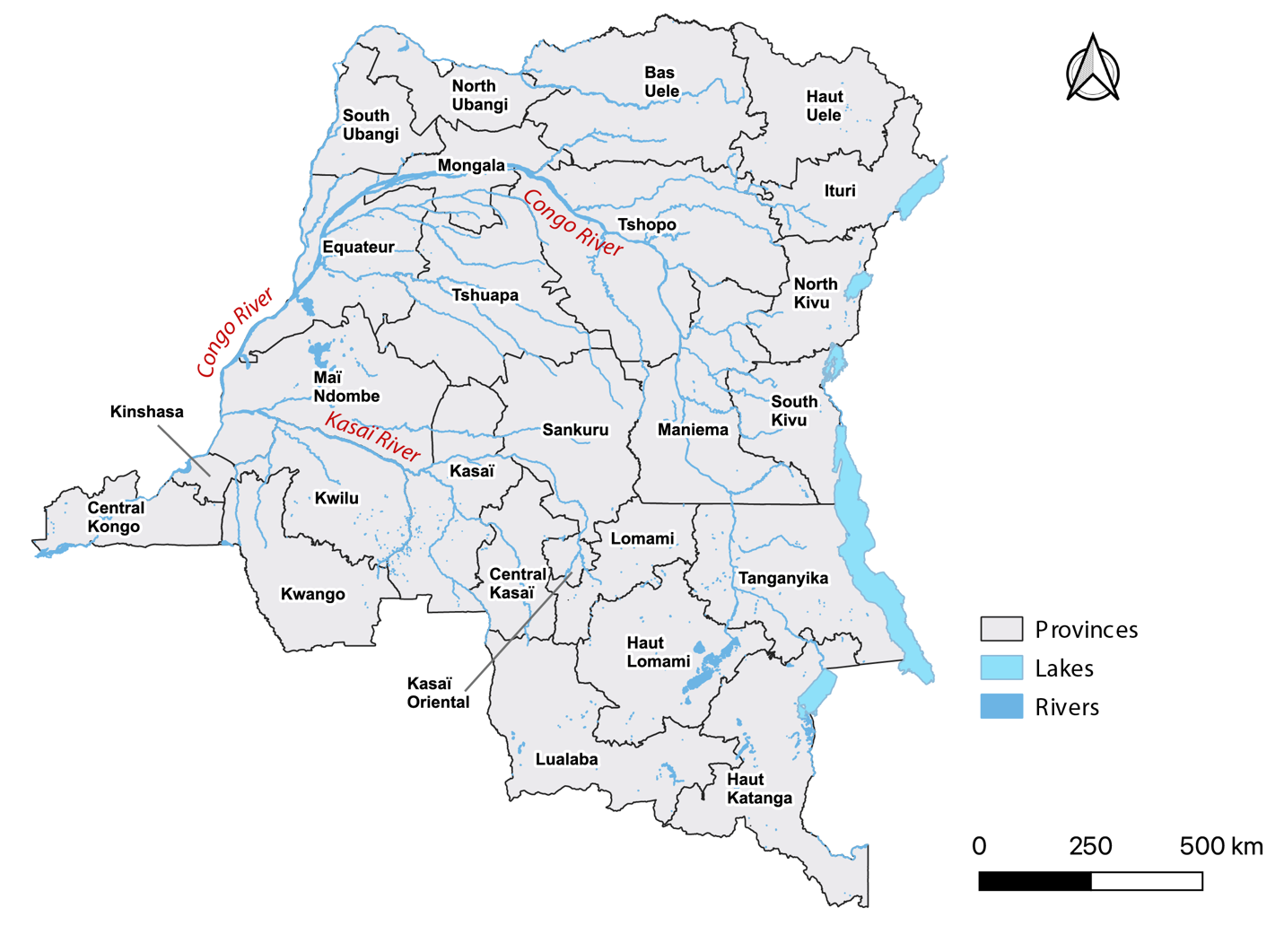


**Additional file 1.** **Context map of the Democratic Republic of the Congo.** Provinces are labeled in bold. The Congo and Kasaï Rivers are indicated in red italics.
