# Supplementary figures and images for: Spatiotemporal dynamics of cholera hotspots in the Democratic Republic of the Congo from 1973 to 2022
Source: BMC Infect Dis. 2024 Mar 28;24:360. doi: 10.1186/s12879-024-09164-9 (PMC10976723; doi:10.1186/s12879-024-09164-9)

Additional file 2


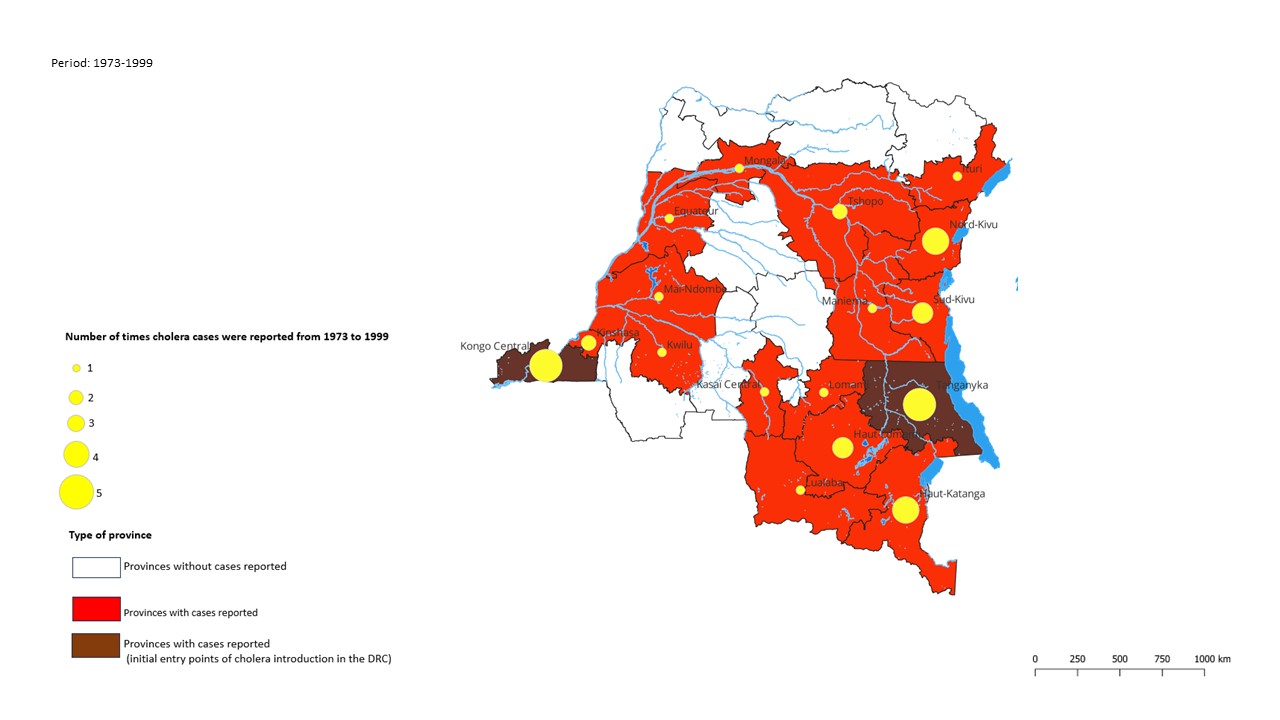
***
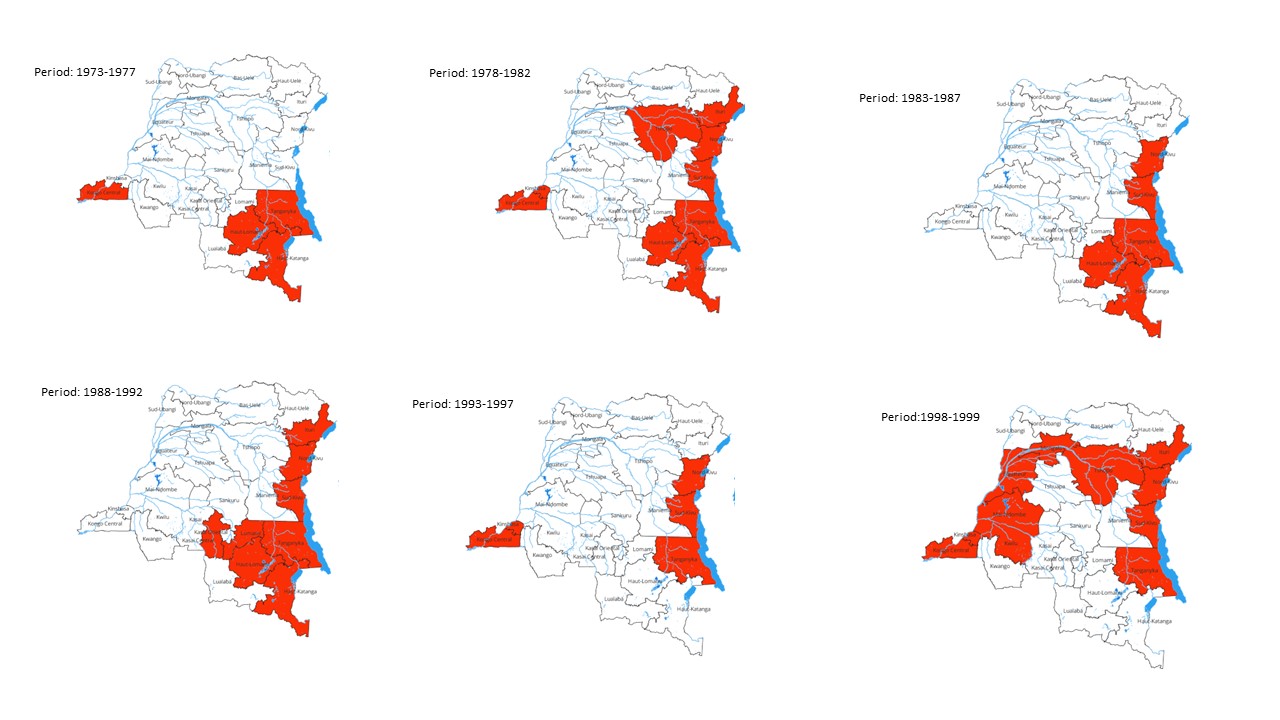
***

**Additional file 2: Provinces reporting cholera cases in the DRC from 1973 to 1999.**

Supplement: Supplementary file 2 — Supplementary Material 2. [file 12879_2024_9164_MOESM2_ESM.docx]
